# Supplementary material for: Insights into Biochemical Sources and Diffuse Reflectance Spectral Features for Colorectal Cancer Detection and Localization
Source: Cancers (Basel). 2022 Nov 21;14(22):5715. doi: 10.3390/cancers14225715 (PMC9688116; doi:10.3390/cancers14225715)
Supplement: Supplementary file 1 [file cancers-14-05715-s001.zip › cancers-1989707-supplementary.pdf]

Supplementary Material

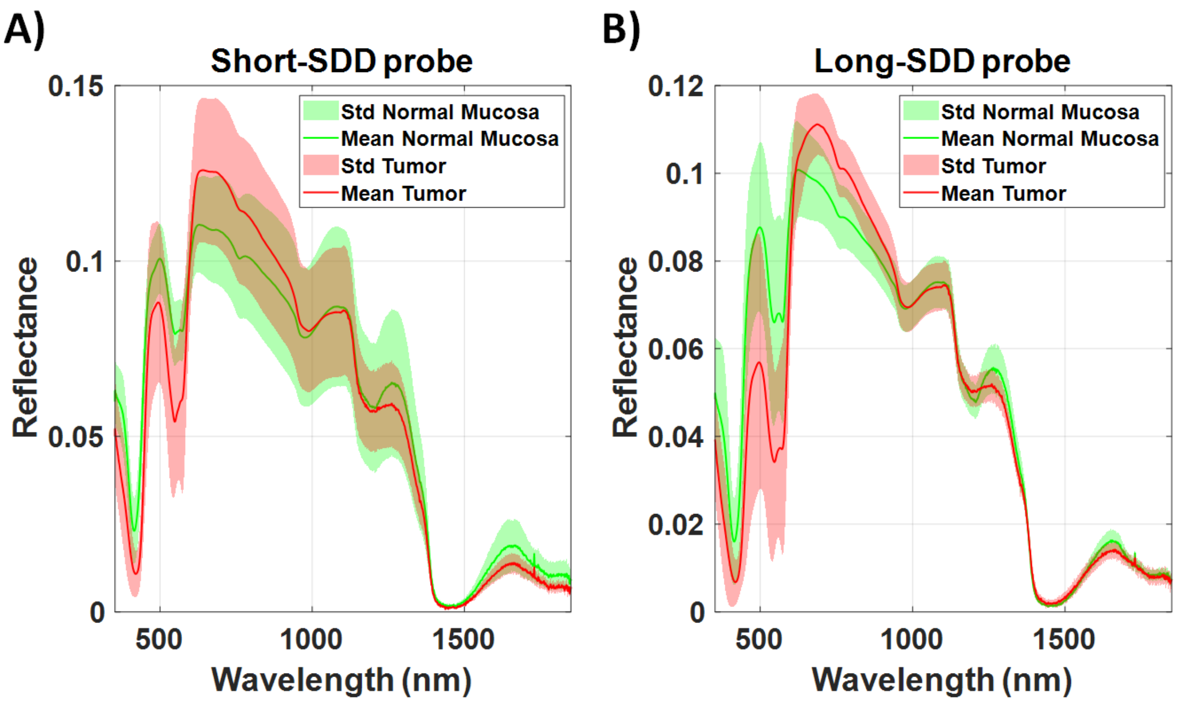

Figure S1. Mean and standard deviation across broadband reflectance spectra of all measured locations of both tumor and normal mucosa measured with (**A**) the short-SDD and (**B**) the long-SDD probe for one patient.
